# Managing Screen Use in the Under-Fives: Recommendations for Parenting Intervention Development

**DOI:** 10.1007/s10567-023-00435-6

**Published:** 2023-05-12

**Authors:** Alina Morawska, Amy E. Mitchell, Leigh R. Tooth

**Affiliations:** 1 Parenting and Family Support Centre, School of Psychology, The University of Queensland, 13 Upland Road, St Lucia, Brisbane, Qld 4072 Australia; 2 School of Nursing, Midwifery and Social Work, The University of Queensland, Brisbane, Australia; 3 Menzies Health Institute Queensland, Griffith University, Brisbane, Australia; 4 School of Public Health, The University of Queensland, Brisbane, Australia

**Keywords:** Screen use, Screen time, Parent efficacy, Parent knowledge, Parenting, Parental interventions, Preschooler, Infant, Socio-ecological

## Abstract

The impact of excessive screen use on children’s health and development is a public health concern and many countries have published recommendations to limit and guide the use of screen media in childhood. Despite this, international studies report that the majority of parents and children do not adhere to screen use recommendations. Existing research aiming to understand children’ screen use has largely focused on older children, and on demographic and structural aspects of the child’s environment. Parents play a central role in determining young children’s screen use and identify numerous barriers to developing healthy screen use practices with their children. However, no clear models exist that incorporate key parenting factors in understanding children’s screen use, which presents an impediment to intervention development. Likewise, while some evidence exists for interventions to improve children’s screen use behaviours, most are focused on older children and parental involvement has generally been limited. In this paper, we overview key factors associated with screen use in young children (< 5 years) and summarise the existing evidence base for interventions designed to support healthy screen use. This paper proposes a conceptual model linking aspects of parenting and the socio-ecological environment to young children’s screen use. Our proposed model could be used to design longitudinal studies of screen use predictors and outcomes, and inform intervention development. Finally, the paper provides key recommendations for future research, intervention development and testing.

## Introduction

The impact of excessive screen use on children’s health and development is a public health concern and many countries have published recommendations in an effort to limit and guide the use of screen media in childhood. Current international and Australian guidelines recommend that infants and toddlers under 2 years old have no screen use at all, and that children aged 2 to 5 years have less than 1 h of screen use per day (Department of Health, 2019; World Health Organisation, 2020). Despite these recommendations, international studies report that only a minority of parents and children adhere to screen use guidelines (McArthur et al., 2022; Rideout & Robb, 2020; Roman-Vinas et al., 2016), and the majority of Australian children under age five have more screen time than guidelines recommend (Chandra et al., 2016; Tooth et al., 2019). Infants and toddlers under age two in Australia average 14 h of screen use per week (i.e., 2 h per day) and children aged 2 to 5 years average 26 h of screen use per week (i.e., 3–4 h per day) (Rhodes, 2017).

Early childhood is a critical time in which family health behaviours and routines are established (Hamilton et al., 2016), and children’s earliest screen use experiences are often formative for later childhood and beyond (Brown & Smolenaers, 2016; Hingle et al., 2010; Hodges et al., 2013). For example, greater exposure to screen media in early childhood has been linked to greater screen use in later childhood (Xu et al., 2016). Children under five years of age spend around 50% of their time engaged in sedentary behaviour (Pereira et al., 2019, 2021), and sedentary behaviours track over time across infancy (Meredith-Jones et al., 2019) and from childhood to adolescence (Biddle et al., 2010), and increase as children age (Kontostoli et al., 2021; Lauricella et al., 2015).

“Screen use” is defined as the time spent viewing or interacting with television, DVDs or videos, computer or electronic games, and smartphones or tablets (Birken et al., 2011), although more recently there have been calls to focus more on the behaviours screens are affording rather than the specific features or devices being used (Kaye et al., 2020). Screen technology is “vastly outpacing research” (Madigan et al., 2020) and there is a relative paucity of research in children under age five relative to research on older children, particularly in the context of mobile devices (Brown & Smolenaers, 2016; Myruski et al., 2018). Much of the research in this area has focused on establishing and identifying factors associated with quantity of screen use, although quality of content is also noted as an important factor. The bulk of the literature centres around television use specifically, with studies of mobile device use only recently emerging (Braune-Krickau et al., 2021; McDaniel & Radesky, 2018, 2020). However, given that screens are a ubiquitous and increasingly essential part of our lives, it is perhaps more important to consider screen use from a *self-regulatory* perspective, where the aim is for adults and children to use screens in a way that is limited, autonomous, skilled, and critically informed. Indeed, research needs to move beyond simply quantifying screen use and its associated effects to better understanding the function of different types of screen use for both parents and children (e.g., Elias & Sulkin, 2019).

In this paper, we briefly examine the effects of screen use on young children under age five, describe the existing evidence for factors associated with screen use, discuss existing models explaining the variables influencing young children’s screen use, and propose a different model that may better capture the complex, inter-connected range of factors that influence child development, and thus have greater capacity to inform the design of interventions that are effective in influencing children’s and families’ screen use. We also summarise the current approaches to assessment of screen use and intervention approaches designed to limit children’s screen use. Finally, we provide a set of recommendations for future research and practice.

## Effects of Screen Use

The effects of screen use have been examined in numerous studies and collated in several systematic reviews (e.g., Hood et al., 2021; LeBlanc et al., 2012; Madigan et al., 2020; Ophir, Rosenberg, & Tikochinski, 2021; Stiglic & Viner, 2019). Overall, reviews have generally found evidence of harm associated with child and adolescent screen use (e.g., Madigan et al., 2020; Stiglic & Viner, 2019), but some empirical studies have found limited effects (Przybylski & Weinstein, 2019) and many of the studies included in these reviews are of low methodological quality (Ophir et al., 2021). Controversies around the effects of screen time have also been noted (Browne et al., 2020; Kaye et al., 2020; "Screen time: how much is too much?," 2019), due to the lack of studies clearly demonstrating causality relating to the effects of screen use. This points to the need for more rigorous, longitudinal studies which take into account the current pattern of device use rather than focusing simply on the amount of time devoted to screen use. It is worthwhile to note, however, that much of the debate surrounding the benefits or harms associated with screen use centres on older children and adolescents, rather than younger children. Developmentally, this is an important distinction as screen use is likely to serve some similar (e.g., entertainment) but also different (e.g., peer relationships) functions at different stages of development, which may impact on the relative risks and benefits of screen use. For example, while for adolescents engaging with their peers via devices may be normative and common and potentially beneficial, this is unlikely to be the case for a toddler or preschooler, thus highlighting the importance of considering the different effects of screen use in different developmental periods.

In terms of young children specifically, a systematic review of 21 longitudinal and experimental studies with children under the age of five found that greater television viewing was associated with poorer psychological health and cognitive development and greater adiposity (LeBlanc et al., 2012). There was no evidence that television viewing was beneficial for improving children’s psychosocial health or cognitive development (LeBlanc et al., 2012). Furthermore, there was some evidence of a dose–response relationship, with greater exposure associated with worse child psychosocial and cognitive outcomes (LeBlanc et al., 2012; Zhao et al., 2018). Numerous studies have examined specific outcomes associated with screen use in young children. For example, more screen time has been associated with less sleep in 4-month-olds (Ribner & McHarg, 2019) and 2- to 5-year-olds (Xu et al., 2016), and greater total and evening screen time is associated with poorer sleep in infants and toddlers (Janssen et al., 2020). Detrimental associations have also been demonstrated between screen time and inattention (Tamana et al., 2019), poorer inhibition (McHarg, Ribner, Devine, Hughes, & Team, 2020), and a number of other developmental outcomes (Madigan et al., 2019). For example, greater screen use is associated with poorer language outcomes in toddlers (Operto et al., 2020), preschoolers (Stockdale, Holmgren, Porter, Clifford, & Coyne, 2022) and, more broadly, in children under 12 years of age (Madigan et al., 2020).

Some research has shown that aspects of parenting and the family environment can mediate the effects of screen time on children’s psychosocial, behavioural and developmental outcomes. For example, parents choosing better quality programming for their children (educational content) and co-viewing (with caregivers) are associated with better language outcomes for children (Madigan et al., 2020). Similarly, while more screen time during infancy was associated with poorer cognitive, language and motor development in the preschool stage, these effects were mediated by negative (non-authoritative) parenting behaviours (Supanitayanon et al., 2020). Likewise, negative psychosocial outcomes of excessive screen time in preschool children were mediated by changes in parent–child interactions (Zhao et al., 2018). These studies point to the crucial importance of exploring the role of parents in young children’s screen use. Ultimately, it is the responsibility of parents to ensure that young children develop healthy screen use habits, because young children are not capable of regulating their own screen use; nor should we expect them to be capable of managing this emerging skill. Thus, to understand the complex social and environmental factors that determine young children’s screen use we must consider parents’ perceptions and behaviours.

## Parent Perceptions of Young Children’s Screen Use

Screen use is one of the most common areas of concern for parents of young children (Rhodes, 2017; Wartella et al., 2014). Nevertheless, parents are ambivalent about screen use (Teichert, 2020) and limiting screen time (Hamilton et al., 2015; He et al., 2005; Jordan et al., 2006; Lauricella et al., 2015). Many parents hold mixed views about the educational potential of screen media. For example, in a nationally-representative survey conducted in the United States, 67% of parents said that their children’s screen media use helps their learning, yet 76% also agreed with the statement, “The less time kids spend with screen media, the better off they are” (Rideout & Robb, 2020). Furthermore, while parents often ascribe educational value to screen use (Reddan, Morawska, & Mitchell, 2022), they report that their children mostly use screens for entertainment, and fewer than one in five mostly use screens for educational purposes (Graham & Sahlberg, 2021).

Parents identify concerns around the negative impacts that screens may have on their young children, including over-reliance on screens, poorer social skills, negative impacts on cognitive and physical development, accessing inappropriate content, displacing other types of play and activity, oppositional behaviour, lack of physical activity, reduced connection to nature and sleep problems (Dardanou et al., 2020; O’Connor & Fotakopoulou, 2016; Rhodes, 2017). On the other hand, parents also see children’s screen use as important for their cognitive and educational development and a necessary skill in modern society (Reddan et al., 2022), as a way of learning new skills, as something that keeps children occupied while parents complete other tasks, as a reward for good behaviour, and as a tool to manage children’s behaviour while in public (Dardanou et al., 2020; O’Connor & Fotakopoulou, 2016; Rhodes, 2017). Parents report advantages of limiting screen time for children, including more physical activity, better wellbeing, and more creativity, but also report disadvantages including problem behaviour and parent–child conflict (Hamilton et al., 2015).

Although parents are aware of screen time guidelines broadly, their knowledge about these tends to be inaccurate (Hamilton et al., 2015) and they typically experience multiple barriers to following screen time guidelines with their children (Hamilton et al., 2015; Reddan et al., 2022). For example, Brown and Smolenaers (2016), in a qualitative study of parents of 0- to 2-year-old children, found low levels of awareness of screen time guidelines, that guidelines were perceived to be unrealistic, and that parents were confused about how to measure how much screen time their child was actually having (e.g., parents did not count their own smartphone use in the presence of their child as screen time). Parents report greater perceived behavioural control in relation to encouraging children’s physical activity compared to reducing children’s screen time after exposure to both physical activity and screen use guideline messaging, which has important implications for parents’ intentions to follow recommendations (Jarvis et al., 2021). Moreover, parents of infants are more confident in their ability to manage children’s physical activity and screen time compared to parents of preschool-aged children, suggesting that screen management skills and strategies should be provided to parents early in their child’s development to support later skill building (Hesketh et al., 2012).

## Factors Associated with Young Children’s Screen Use

Numerous variables have been explored in attempting to understand the influences on children’s screen use. These include demographic, home environment and parenting factors. A number of parent (e.g., parent education) and child (e.g., age, number and ages of siblings) demographic factors have been linked with young children’s screen use (Chandra et al., 2016; Corkin et al., 2021; Duch et al., 2013; Goh et al., 2016; Hoyos Cillero & Jago, 2010; Paudel, Jancey, Subedi, & Leavy, 2017; Poncet et al., 2022; Tooth et al., 2019; Yu & Baxter, 2015), however, these are variables which are not easily modifiable, and while they may help in identifying children at risk of high or problematic screen use they have limited utility in intervention design. Compared to research with older children and adolescents, there is relatively limited information on factors associated with young children’s screen use beyond demographics (Elias & Sulkin, 2019; Paudel et al., 2017) and modifiable factors remain to be clearly described.

Drawing on the relatively small body of work examining other factors associated with young children’s screen use, structural aspects of the home environment (e.g., electronic equipment in bedrooms, rules around screen time, type of screen use, availability of outdoor play space and equipment) have been associated with screen use in early childhood (Chandra et al., 2016; Corkin et al., 2021; Goh et al., 2016; Tooth et al., 2019; Yu & Baxter, 2015). In addition to parenting perceptions about screen use, as described previously, there are also a number of parenting practices and parental knowledge, attitudes and motivations that are associated with screen use in very young children.

Parenting practices include how parents manage their own screen time and the screen time of their children. Parental screen time is strongly associated with child screen time (Bleakley, Jordan, & Hennessy, 2013; Lauricella et al., 2015), supporting a modelling pathway (Goh et al., 2016; Tang, Darlington, Ma, Haines, & on behalf of the Guelph Family Health, 2018). However, while parents may be aware of the impact of their own screen use on their children (Graham & Sahlberg, 2021; Radesky et al., 2016), they often lack motivation and skills to change their own habits (Reddan et al., 2022). Most adults believe that older children’s screen time should be limited, however do not adhere to limits themselves (Minges et al., 2015; Schoeppe et al., 2016). Thus, parents’ own use of screens, their knowledge and attitudes towards that screen use, their motivations to change and their capacity to self-regulate their own screen use are important to understanding child screen use.

In terms of knowledge, better knowledge of screen time recommendations has been associated with less child screen use (Miguel-Berges et al., 2019). However, parent knowledge about screen use is not necessarily accurate. For example, Hinkley et al. (2017) found that while the majority of mothers believed screen time was detrimental to their children's physical wellbeing, most did not believe that screen time was harmful to children's cognitive and social wellbeing outcomes.

Parent attitudes towards screens likewise have an important role in children’s screen time (Lauricella et al., 2015). Parental perceptions of technology have been shown to be important predictors of school-aged children’s screen time (Sanders et al., 2016) and parental intentions about limiting screen time (Russell, Huber, & Morawska, 2022). For parents of younger children, attitudes toward screen time predict intentions to reduce preschoolers’ screen time, and parents’ beliefs about the positive and negative effects of screen time are associated with intentions and behaviour to limit screen time (Hamilton et al., 2016). Likewise, children whose parents hold negative perceptions about limiting screen time are more likely to exceed screen time recommendations (Miguel-Berges et al., 2019).

The practices parents implement around screen use also affect their ability to implement screen use guidelines. In particular, research has examined the role of monitoring, limiting and co-viewing with young children. In general, parental monitoring (Downing et al., 2015; Lampard et al., 2013; Neshteruk et al., 2021; Tang et al., 2018; Thompson et al., 2015) and limit setting (Downing et al., 2015; Hoyos Cillero & Jago, 2010; Lampard et al., 2013; Neshteruk et al., 2021; Tang et al., 2018; Thompson et al., 2015) are associated with less screen use. In contrast, co-viewing (Thompson et al., 2016) and using screen time to control child behaviour (Tang et al., 2018; Thompson et al., 2016) are associated with greater screen use. Most recently, research has indicated that use of mobile devices to calm young children is associated with poorer self-regulatory skills in children over time, highlighting the importance of examining specific parenting strategies, the reasons why parents use particular approaches, and effects on children (Radesky et al., 2023).

Less research has examined broader parenting styles among parents of young children as potential correlates of children’s screen time. Authoritarian and permissive parenting styles are associated with more screen time for school-aged children (Langer et al., 2014), while in adolescence, positive parenting is associated with less problematic social media use (Geurts, Koning, Vossen, & van den Eijnden, 2022). Positive parenting has been associated with less screen time overall among young children (Schary et al., 2012; Veldhuis, van Grieken, Renders, HiraSing, & Raat, 2014).

Parental efficacy in relation to screen use has also been identified as an important predictor of screen use. Low parental self-efficacy has been associated with more screen use in a number of studies (Chen, Chen, Wen, & Snow, 2020; Halpin et al., 2021). Maternal self-efficacy to limit screen time is inversely associated with screen time exposure in the under-fives (Campbell et al., 2010) and infants (Hnatiuk et al., 2015).

Beyond the immediate parenting context and practices, the broader parenting and family environment have also been shown to contribute to children’s screen use, although the evidence here is more limited. For example, maternal distress has been associated with greater screen use by young children (Duch et al., 2013; Hoyos Cillero & Jago, 2010; McDaniel & Radesky, 2020), while the experience of fewer life pressures by parents is associated with lower screen use (Lampard et al., 2013). Disagreement between parents about children’s screen use has also been noted as a common issue (Jago et al., 2016), and use of devices can impact the co-parenting relationship (McDaniel & Coyne, 2016).

In summary, numerous correlates of young children’s screen time have been identified, with demographic factors associated with screen use in many studies. As noted earlier, demographic factors are largely non-modifiable, thus do not necessarily inform intervention design. Modifiable factors, such as structural aspects of the home environment along with parental modelling of screen use appear to be linked to young children’s screen time and are clear targets for intervention. Some specific parenting practices (e.g., monitoring and limit setting) as well as parental self-efficacy also appear to be important and are potentially modifiable. Importantly, the correlates identified in the literature are largely confined to children’s time in using screens and are mostly focused on television use. Given the changing nature of the screen media environment, it is important to understand the patterns of children’s screen use, instead of simply trying to count the numbers of hours of screen time they have.

One of the key weaknesses of work in this area is the lack of a systematic, integrated approach to understanding young children’s screen use. While numerous studies have examined factors influencing children’s screen time, they have generally not explored the complex inter-relationships between parental modelling, parental beliefs and attitudes and how these affect their parenting practices in relation to children’s screen use. Nevertheless, parents are the gatekeepers and mediators of young children’s screen use and have control over the amount of time children spend on electronic devices, the circumstances (context) in which children use screens, and the content they engage with (Jago, Edwards, Urbanski, & Sebire, 2013; O’Connor & Fotakopoulou, 2016). Thus, better understanding of parents’ beliefs, attitudes and practices is central to influencing children’s screen use habits.

## An Integrated Model of Factors Influencing Young Children’s Screen Use

Existing models of correlates associated with young children’s screen use have not considered important elements, and are especially lacking a comprehensive focus on the socio-ecology of children’s lives (e.g., Lee et al., 2018). We propose a model that integrates the multiple inter-connected factors within the child’s family environment and include the proximal and distal elements in a child’s socio-ecology that influence screen use. A systematic, integrated approach to understanding young children’s screen use habits needs to consider the numerous interacting influences within their proximal environment (Kalinowski, Xu, & Tekinbas, 2021; Tooth, Moss, & Mishra, 2021). The socio-ecological context of development is a central framework for understanding child development (Bronfenbrenner, 1992; Bronfenbrenner & Morris, 2006), and is useful in explaining how proximal elements (e.g., parents, siblings) and more distal elements (e.g., neighbourhood, media, social policy) in a young child’s socio-ecological system can influence child and family health and health behaviours. For young children, their physical and social home environment is likely to represent the greatest potentially modifiable influence on screen use (Pereira et al., 2021; Rhodes et al., 2020).

Socio-ecological models (Bronfenbrenner, 1992; Bronfenbrenner & Morris, 2006) highlight the social contexts in which the child lives and the frequency with which specific interactions take place. Media use is highly prevalent and common in children’s home environments, and so has the potential to exert a considerable effect on children’s development via its impacts on family interactions, activities and behaviours. Similarly, in social cognitive models, children not only learn by watching others (i.e., what parents are modelling via their own screen use) but also through recurrent, reciprocal patterns of parent–child interactions which are influenced by parental beliefs and self-efficacy (Bandura, 1986).

**Fig. 1:**
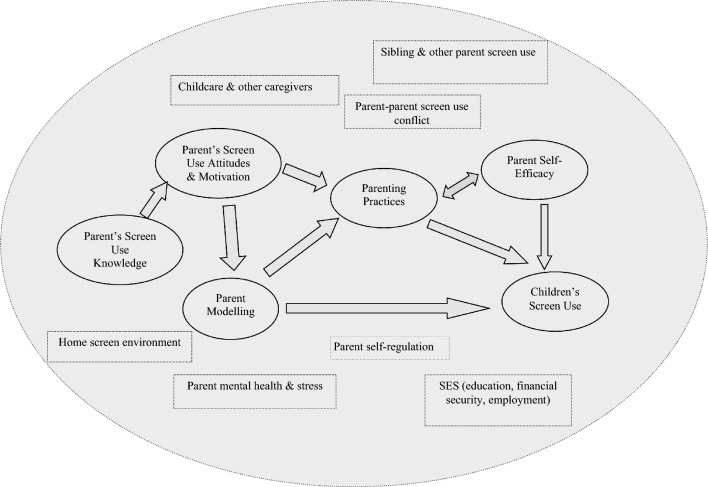
An integrated model of parenting influences on child screen use

We have proposed an integrated conceptual model that brings together what is currently understood about the proximal (primarily socio-ecological) influences on young children’s screen use (Fig. 1). Screen use in this model is defined broadly, to include both time spent using screens but also the pattern and function of screen use. Issues around assessment of screen use are described in more detail in the next section. Our proposed model builds upon and extends previous work on the correlates of screen time by taking a broader and more comprehensive socio-ecological perspective to understanding how child-, parent- and family-level factors may combine to influence children’s screen use. For example, while some previous works have included elements of the child’s family ecology (e.g., Lee et al., 2018), these have not comprehensively integrated the multiple factors identified in the literature as influencing child screen use. Our model places parenting practices at the centre of an array of potentially complex direct and indirect relationships, reflecting the key importance of parenting practices to screen use within the early childhood context. Around this central focus, other sociodemographic and environmental factors relevant to the young child’s socio-ecological context are incorporated, including parental knowledge, attitudes, motivations, and confidence which are recognised as potent shapers of parents’ own behaviours and parenting practices.

Our model posits that three central aspects of parenting play a direct role in influencing children’s screen use: parental modelling, parenting practices and parental self-efficacy. These variables have been repeatedly linked to children’s screen use by prior research, as described earlier. For example, there are clear, strong links between parental screen use and child screen use (Bleakley et al., 2013; Lauricella et al., 2015). Similarly, several specific parenting practices have also been linked to child screen use (Radesky et al., 2023; Tang et al., 2018; Thompson et al., 2016). Parenting practices and modelling are, in turn, influenced by parental attitudes and beliefs regarding screen use (Hamilton et al., 2016; Miguel-Berges et al., 2019). For example, a parent who has positive attitudes and expectations about screen use is likely to model more screen use themselves, engage in parenting practices that enable more child screen use (e.g., fewer rules around screen use) and feel more confident in their ability to manage their child’s screen use.

Beyond parental modelling, practices and self-efficacy, numerous other parent and familial variables are likely to moderate these effects. For example, a parent’s capacity to model appropriate screen use may be moderated by their own self-regulatory capacity and the extent to which they experience stress or mental health problems (McDaniel & Radesky, 2020). Similarly, a parent’s ability to engage in positive parenting practices regarding screen use may be affected by the extent of agreement from their partner or other family members about screen use (Jago et al., 2016). Likewise, the presence of siblings, particularly older siblings, may create additional models of screen use behaviour, contributing to family conflict about screen use and affecting the parent’s sense of self-efficacy relating to managing a younger child’s screen use. We propose that our model be used to inform research aiming to understand screen use in young children and interventions designed to support parents and families in making conscious, considered, evidence-informed decisions about their screen use practices.

One of the key aspects to consider in terms of children’s screen use is the extent to which parents are making reasoned decisions about when, why, and how their child uses screens. For example, some authors have noted that parents are not, in fact, making specific choices or decisions about smartphone use with infants as they see it as a ubiquitous and utilitarian part of life (Golden, Blake, & Giuliano, 2020). Similarly, the issue of parents’ self-regulation in relation to their own mobile device use has also been noted as important to explore (Golden et al., 2020). While most research on children’s screen use to date has focused on the amount and the content of screen media use, much less has attempted to examine or explain parental decision making around screen use. Given that screens are ubiquitous and increasingly essential to everyday life, our understanding of children’s screen use needs to go beyond simply identifying risk factors for high screen use and attempting to reduce screen time. Instead, we need to focus more on understanding when, why and how screens are being used, identifying the modifiable influences on children’s screen use and, importantly, designing interventions that assist parents to be able to make conscious, considered decisions about their own and their child’s screen use.

## Assessment of Screen Use

One of the current challenges in the field is ensuring adequate assessment of parents’ and children’s screen use (Barr et al., 2020; Byrne, Terranova, & Trost, 2021). While child or parent self-report and parent proxy-report have been the most common methods of assessment in research to date, studies of self-report of screen use suggest low validity (Boase & Ling, 2013), where adults tend to under-report their use of television (Clark et al., 2009) and smartphones (Lee et al., 2017). Recent studies of parents suggest there is poor correspondence between caregiver self-report and objective measures of child and parent screen use (Barr et al., 2020; Parker et al., 2022; Radesky et al., 2020). Current work on objective measures of screen use holds promise in terms of obtaining more accurate measures of child exposure (e.g., Radesky et al., 2020; Vadathya et al., 2022), however, given the number of devices a child may access in the home and in the community, both actively and passively, the task of estimating screen exposure is complex. The key is ensuring that young children’s exposure to all forms of screens is adequately recorded, including background television and incidental smartphone viewing such as when the parent is using their phone while their child sits in their lap. Given the ubiquity of screens, capturing the variety, type and extent of exposure is important to understanding the influences on children’s screen use and the extent to which interventions can result in changes to screen use patterns. Importantly, assessment should consider not only the time spent using various devices, but also the content accessed, the purpose of the screen use and the extent to which the screen use is consistent with parental goals for their child.

## Interventions to Change Young Children’s Screen Use Practices

Given evidence of the considerable negative influences of excessive screen use on children’s development (Stiglic & Viner, 2019) and the important role of parents in influencing children’s screen use, interventions that directly target parents may be a promising avenue for supporting the development of healthy screen use habits with young children. The home environment is an important target for change in children’s sedentary behaviour, and aspects of parenting and parental attitudes have been identified as key intervention targets. Interventions have shown sustained effects on parents’ knowledge about screen time (Delisle Nyström et al., 2021), however, interventions focusing on knowledge of behaviour alone are not sufficient for change (Azevedo et al., 2019). Similarly, while technological solutions to manage screen use are available, these are fraught with security and privacy concerns (Ali et al., 2021) and have the potential to negatively affect the parent–child relationship (Ghosh, Badillo-Urquiola, Guha, Jr, & Wisniewski, 2018).

Results from systematic reviews and meta-analyses investigating the effectiveness of interventions aimed at decreasing young children’s screen use demonstrate modest reductions in screen use and sedentary time (Downing, 2018; Krafft et al., 2021; Lewis et al., 2021; Martin, Bednarz, & Aromataris, 2021; Schmidt et al., 2012; Zhang et al., 2022) although findings have been mixed (Wahi et al., 2011; Wu, Sun, He, & Jiang, 2016). An umbrella review of systematic reviews of screen time interventions reported that those targeting children and adolescents were generally effective although effects were small (Nguyen et al., 2020). Importantly, programs delivered in home- or clinic-based settings, rather than child-directed interventions in educational or school-based settings, appear to yield the greatest improvements in terms of screen use reduction, highlighting the importance of taking parental perspectives and involvement into account in intervention design and delivery (Schmidt et al., 2012).

The existing systematic reviews provide some evidence as to what intervention elements and approaches may be most effective. For example, briefer interventions that focus specifically on screen time modification appear to be more effective than longer, multi-component ones (Jones et al., 2021; Martin et al., 2021). The notion that brief, focused interventions may be more effective than longer, more complex ones is also supported in the broader parenting intervention literature (Bakermans-Kranenburg, van, & Juffer, 2003; Leijten et al., 2018; Pinquart & Teubert, 2010). Goal setting, planning and self-monitoring appear to be associated with greater reductions in children’s screen time compared to other behaviour change techniques (Jones et al., 2021). Other promising intervention strategies that have been associated with better outcomes for young children include focusing on changing parenting perceptions, substituting screen time with other behaviours, and parental modelling (Lewis et al., 2021). Focusing on shared family goal setting regarding screen use within a self-regulatory framework (Owenz & Fowers, 2020) may be one way to assist families to engage in healthier screen use practices.

The systematic reviews also highlight several gaps in relation to screen time interventions. Firstly, most interventions to date have focused on reducing screen use, rather than on developing healthy screen practices (Lewis et al., 2021). As noted earlier, this is an important issue given that screens are ubiquitous and increasingly essential to functioning in society. Secondly, very few studies that had tested interventions to improve screen use in younger children were identified, even though the preschool period was noted as an opportune timepoint for intervention and early patterns of screen use are likely to set the foundation for later habits. Furthermore, few interventions targeted parents specifically, in contrast to the emerging evidence suggesting that such interventions are more likely to influence child screen use than those targeting the child directly. This is especially the case in the context of early childhood, where children are not able to make informed decisions about screen use and where parents are central to promoting children’s developing self-regulatory skills.

There is emerging evidence for parenting intervention to reduce screen use by young children (Adams et al., 2018; Sanders et al., 2018); however, engagement into parenting interventions remains a significant problem (Ingoldsby, 2010; Piotrowska et al., 2017) and few studies have empirically examined parent engagement strategies (Gonzalez et al., 2018). Given parents’ high screen use and their ambivalent attitudes about children’s exposure to screens, engaging parents into such interventions is likely to be particularly challenging. Hence, it is critically important to identify parental attitudes, beliefs and motivations relating to screen use which play a role in parental decision making around their young children’s screen use. Our model captures all of these elements within the child’s family environment and thus has the potential to inform more integrated interventions which address the factors necessary to influence family screen use practices.

## Recommendations and Future Directions

Parents are their child’s first teacher, and most are keen to gain knowledge during their child’s early development (Baker et al., 2020); yet existing services are not evidence based and have not been effective in changing population level screen use practices among young children or any other age group. Even when parents have accurate knowledge about lifestyle factors that contribute to the long-term health of their children, they may face barriers (such as their child’s refusal or lack of cooperation) in implementing the recommended strategies with their children (Baker et al., 2020); knowledge alone is therefore not enough to establish healthy habits. For example, parents’ ability to incorporate healthy habits (e.g., appropriate quantity and quality of screen time) into their child’s daily routine, manage their child’s resistance to health activities (e.g., when switching off screens), place limits around their child’s behaviour (e.g., screen use at bedtime), and role-model healthy behaviours (e.g., limiting screen use during mealtimes) are all important aspects which may influence parents’ ability to meet child health recommendations and, ultimately, improve child health outcomes. In this section we outline some key recommendations and future directions.

While research with older children has examined the influence of social norms, social messaging and peer influences on screen use, the socio-ecological influences on the day-to-day behaviour and experiences of younger children are quite different. Thus, future research needs to replicate, extend or adapt research questions, study designs and methodologies for use with families of young children from birth to preschool age to better understand whether and what findings from studies with older children hold true in early childhood, and to identify additional factors that may be specific to the early childhood context. This should include high-quality longitudinal studies to enable identification of causal (and not just correlational) relationships to build a valid and reliable evidence base that can be used to develop targeted interventions that are tailored to the socio-ecological systems in which young children and their families operate.

Since parents have arguably the strongest influence on young children’s health behaviours, future research should incorporate comprehensive assessments of parents’ beliefs, perspectives, knowledge, attitudes and behaviours to improve our understanding of how, when, and why these factors influence screen use in early childhood. Existing research has revealed considerable variability in parents’ knowledge, attitudes and beliefs about children’s screen use, but relatively little is known about how these factors operate separately or together to influence parents’ own day-to-day screen use and that of their young children. In particular, more research is needed to examine parents’ understandings of the family and structural factors within their lives that are associated with their own and their children’s screen use, and to better understand how and why parents manage screen use within their own family context. For example, systematic reviews indicate that any benefits that children may derive from screen use are confined to later childhood and adolescence (e.g., Hood et al., 2021; LeBlanc et al., 2012; Madigan et al., 2020; Ophir et al., 2021; Stiglic & Viner, 2019); however, if parents believe that their young children will likewise benefit, this may affect the way they manage their child’s screen use. Thus, more research is needed to examine parents’ attitudes and understanding of the risks, benefits, and determinants of screen use in their very young children, and how these differ from older children.

Likewise, we need a better understanding of the important within-family interactions that govern young children’s screen use (Thompson & Tschann, 2020). Although some research has begun to examine the direct and mediating influences of parent–child interactions and relationships on young children’s screen use (e.g., Elias & Sulkin, 2019; Lederer et al., 2022; Stockdale et al., 2020; Supanitayanon et al., 2020), other factors such as inter-parental agreement about household rules and expectations, and sibling interactions and influences, are likely to be important and require further exploration. This is especially important given the evidence that young children with older siblings have greater screen use compared to young children without older siblings (Tooth et al., 2022). Research is needed to disentangle the complex relationships between parents’ knowledge and attitudes, parenting practices, sibling influences, and other within-family interactions and processes that influence children’s screen use, particularly in families with children of different ages or where age differences between children are greater.

Furthermore, research should encompass a more comprehensive systematic approach to explaining the influences on young children’s screen use, in order to inform effective intervention targets (e.g., Lee et al., 2018). Testing integrated models, such as the one we have proposed, using longitudinal study designs can help disentangle the complex inter-relationships between parenting beliefs, attitudes, motivation and practices to help identify key modifiable factors, which can serve to inform intervention development. Similarly, systematic reviews of the literature to examine the relative importance of factors associated with screen use across childhood are needed to inform intervention design.

Development of appropriate measurement tools to assess parent and child screen use is a central challenge for researchers in the field (Byrne et al., 2021; Thompson & Tschann, 2020). While some progress in this area has recently been made in terms of gaining more comprehensive assessments of children’s screen use and exposure (e.g., Barr et al., 2020), assessment of parental attitudes, decision making and goal setting around screen use are limited (Byrne et al., 2021). As noted earlier, we need to go beyond simply assessing how much screen time parents and children are engaging in, and instead focus more on the extent to which screen use is autonomous, skilled, and critically informed. Given that screens are now ubiquitous, the central issue is no longer how *much* screens are being used, but *how* they are being used, in what context and under what circumstances, and the extent to which this is consistent with parents’ goals for themselves and their children. Developing assessment tools which take into account these more nuanced aspects of screen use will better inform our understanding of screen use across the lifespan, but also assist in intervention evaluation, particularly if the goal of interventions is not simply to reduce screen time but to assist in the development of more self-regulated screen use habits.

Finally, intervention research is needed to design and test effective approaches to ensuring healthy screen use practices. Existing evidence provides some direction for intervention design, suggesting that brief, parent-focused interventions are likely to be important and need to go beyond simply telling parents about screen use guidelines and finding ways to overcome barriers to implementing such guidelines. For example, a harm reduction approach to infant screen use has been described which incorporates a number of specific recommendations for interventions in this developmental phase (Heller, 2021). We need better ways to help parents find ways to more positively integrate the reality of digital screens into family life. This might include ensuring parents keep screen time to moderate levels, that content is safe and developmentally appropriate, ideally designed to promote development, and making sure screen use is integrated within other appropriate developmental tasks. Intervention design and testing should focus on the earliest periods of child development, given that screens are omnipresent and an increasingly integrated part of our lives. Parents should be able to access evidence-based guidance and information that helps them to make informed choices about how they want to use screens and how they want their child to engage with screens.

## Conclusions

Research has already identified relationships between greater screen use and worse health and developmental outcomes for young children, with no evidence of tangible benefits. However, our focus now needs to widen to better understand the effects of content and context, and to disentangle the complex relationships within young children’s social environments that influence how, when and why they use screens. We have proposed an integrated conceptual model that draws together important proximal factors relevant to the early childhood context, identifying parenting practices and related parenting factors as important foci for future work. It provides a foundation for future longitudinal and experimental research to disentangle complex direct and indirect relationships between parents’ knowledge, attitudes, beliefs, and behaviours and young children’s screen use; to understand how child-, parent-, and family-level factors act, individually and in combination, to govern parenting behaviour and young children’s screen use; and to identify the key modifiable factors that can be effectively targeted through intervention to improve screen use behaviours in early childhood. Ultimately, improved understanding of these relationships will support the development of socio-ecologically valid evidence-based interventions to help parents to develop considered, self-regulated and competent screen use practices that are consistent with their goals for themselves and their children and support healthy development in early childhood.
